# A Forensic Tale of Nepal

**DOI:** 10.31729/jnma.3715

**Published:** 2018-10-31

**Authors:** Hemang Dixit

**Affiliations:** 1 Department of Medical Education, Kathmandu Medical College, Duwakot, Bhaktapur, Nepal

**Keywords:** *autopsies*, *Bir Hospital*, *forensic medicine*, *Institute of Medicine*

## Abstract

An account is given of ancient funeral practices and then of the development of forensic medicine from the mid twentieth century. Early forensic practices at Bir Hospital have been recorded and then the subsequent development of the study of forensic medicine in the country has been stated.

## BACKGROUND

One of the earliest visitors to Nepal was Father Greuber, an Austrian Capuchin monk who on his return journey from China to Europe, passed through Nepal. He was much disturbed by what he saw and went on to write:

‘If a sick man is near to death and no further hope of his living is entertained, they take him outside away from his house into the fields, and there throw him into a ditch already full of dying men. He there remains exposed to the inclemencies of the weather, without consolations of religion nor pity they leave him to die; afterwards his corpse is given to birds of prey, wolves, dogs, and other similar beasts to eat. They are convinced that the only monument of a glorious death is to find a resting place in the belly of living animals'.^1^

## INTRODUCTION

In my younger days I was an avid reader of books that may be labeled as ‘Who Dun It'. My favourite was Agatha Christie and during the time that I was doing the medical course I remember finishing I think it was three paperbacks over a period of 24 hours. I would not recommend doing so for after reading a number of books by the same author over a short period one gets to know a little bit about the thinking process and can perhaps guess as to who the culprit or murderer is. Being a fan of Agatha Christie I was thrilled to meet her at the Royal Nepalese Embassy in the nineties sixties and learn that she had visited Nepal. I asked her then if she would write a crime novel set in Nepal. She replied, ‘No’. No. Nepal is such a lovely country; I would not like to set a murder there!”

During the time that I was studying medicine we had a lecture by a well-known pathologist John Dickenson Carr. He gave a talk at Charing X Hospital Medical School, where I was studying on ‘The Diseases of the Kings and Queens of England.’ This too whetted my appetite regarding forensic medicine for the reason that Dr. Carr besides being a Home Office Coroner was a well known crime fiction writer.

Our usual practice then would usually be to attend post mortems done by a pathologist or by a home office coroner - a pathologist who was officially designated so for the purpose, during the daily lunch break. House officers or registrars under whose care the patient had died had to attend. In the 1950s it was almost customary to do autopsies on those patients who died within twenty-four hours of admission in hospital. I feel that it should be made obligatory in Nepal to do autopsies in those cases where the cause of death is in doubt.

My interest in forensic medicine was at first spurred on by my reading of detective fiction. It perhaps increased after coming in contact with the ‘fast track’ book on anesthesia by a certain Richard Ostlere. This medical doctor, who also wrote under his pen name of ‘Richard Gordon’, became a household word in the UK following his “Doctor in the House' and other books and films. He was so successful that he gave up medical practice altogether. He also wrote more serious books of fiction under his pen name on the discovery of anaesthesia and penicillin. His book on Forensic Medicine - The Final Witness is a classic. It should be read by all but unfortunately is out of print.

Another book which I have been recommending to many people is “Echoes of Pain” written by Dr. Ravi Thapaliya, an Institute of Medicine (IOM) product who now lives in Australia. This book is set in Nepal, was published by Sajha Prakashan in 2005 and deals with what a Nepali doctor may have gone through during those times. I have recommended this book to many but it too may be out of print now, its readership being limited in Nepal.^2^

History of Crime by Colin Wilson speaks of the origin of finger printing in Nepal. In prehistoric Babylon it was customary to use fingerprints for business transactions. Chinese records exist showing use of handprints as evidence in burglary investigations during the Qin Dynasty rule (221-205 BC). In 1400 AD in Persia fingerprints were used to identify persons.^3^ However a certain William Herschel, a British Magistrate in Bengal in 1858 noticed the use of hand or fingerprints in legal documents signed by Bengalis. Initially he thought it was superstition but later realized its correctness. A Scottish physician Dr. Henry Faulds later also realized this in 1880 and even asked Charles Darwin to investigate further. Darwin declined and forwarded this question to Galton who then worked on it. Now fingerprinting for identification is an essential part of forensic medicine.^4^

## EARLY FORENSIC PRACTICES

Returning back to Nepal I found out that autopsies at Bir Hospital were generally done by doctors appointed as Police Surgeons on jail duty. My first knowledge about this practice was about 1962 when Mohan Daju, a relative of mine was selected to go and study forensic medicine in India. He would perhaps have been the first doctor in Nepal with this specialty. However, he being the jail doctor was sent in a helicopter flown by a well experienced pilot Captain Randhawa to investigate a plane accident that had taken place at Dhorpatan. They went and came back. The second trip on 24^th^ August 1962 did not have a similar ending. The helicopter crashed and the captain, Dr. Mohan and SP Rameshwar P Upadhaya of Pokhara were all killed. Tragically what would have been an early foray into forensic medicine practice, had Mohan Mani started and completed his chosen area of study, did not become a reality.

The tale of human dissection in Nepal may be said to have started almost a hundred years ago when an interesting post mortem examination or on cutting up of the human body took place. A certain Dr. Raj Krishna Mukerjee had come to Nepal during Bir Shumsher's time. A news item in the then weekly issue of the Gorkhapatra dated 11^th^ Jestha 1985 BS (1928AD) reported that Dr. Mukerjee had dissected dead bodies and showed the various internal organs to the then prime minister and to a group of vaidyas or local practitioners of the healing profession.^5^ Later, during Dev Shumsher short premiership of 114 days, Dr. Mukerjee was appointed a lecturer at the Nepal Medical School. Another point to note is that Dr. Mukerjee then translated the then current edition of Gray's Anatomy (Shareer Tattwa) into Nepali. A copy of this book is in the Madan Puraskar Library at Lalitpur.^5^

In the 1960's and onwards it was customary to have the dead bodies brought to Bir Hospital for post mortems to be done. I was aware of this when I worked in that institution in the late 1960s. The autopsies were conducted under the supervision of the jail doctors, though the person who usually did all the actual carving was the porter attached to the department. This was the practice elsewhere in the country too. It was not surprising to find that during the time of storage of the cadavers in the premises, that some parts of the bodies e.g. the eye or the ear were sometimes surreptitiously taken away by the rodents that were scurrying around nearby. The Police Surgeon or jail doctor at that time was Dr. Dwarika P Manandhar and he was assisted in this job by Dr. MR Baral who was the Resident Medical Officer at Bir Hospital and also looked after patients in the Central Jail. Dr Baral then had to do a number of autopsies during his tenure. Subsequently Dr. Achyut Bahadur Shrestha, who was appointed Police Surgeon, did the autopsies at Bir Hospital.

What is a point to note is that during my period of posting at Bir Hospital, an autopsy of a baby born dead was performed by Dr. Dibya Shree Malla and Dr. Lakshmi N Prasad. The account of this was published in the Journal of the Nepal Medical Association in 1968. The abstract of this, as printed in the Health Sciences Bibliography of Nepal 1950–1977 is as follows:^6^

Case report of a neonatal death with post mortem findings from Bir Hospital. The cause of death was shown to be incomplete expansion of the lungs resulting in respiratory failure. Although histological findings are unavailable, this is a case of pulmonary atelectasis.

It is interesting to note that the Nepal Police Act of 2012 BS (1965 AD) stated that unclaimed bodies found should be handed over to the nearest medical school when in fact no medical school /college existed in the country.^7^ However, such provision is commendable in terms of forward thinking!

Generally the cutting open of the bodies and exposure of the organs was done by the attendant attached to the department who had been directed and had become proficient in the task. About a year back there was a report in a Kathmandu daily newspaper about a Ram Bahadur Damai, a resident of Kushma, Parbat.^8^ His initial appointment had been as a helper at the hospital and had to do the work of cutting and exposing open the bodies for further dissection. He reckoned that over the period of 27 years he had cut open, as many as 2000 bodies. Initially he was not given any extra payment but now gets Rs. 170/- after taxes as remuneration per body. This unseen body of behind the scene workers in the forensic field needs to be appreciated.

When the IOM started the MBBS course, it was but natural that the students had to have instruction in forensic medicine as it was a part of the curriculum. The students who did their medical studies in India had this training but those doing it in China did not have instruction in forensic medicine. The Nepal Medical Council then brought this fact to the attention of the concerned Chinese authorities who, in course of time, rectified it. Initially, the Nepali students who had studied medicine in China, once they came back home had to observe a number of postmortems before they were posted in different parts of the country.

After this, the Institute of Medicine also arranged a number of training sessions in which Dr. Sudhamshu Sharma played an active role in the training of young doctors on forensic medicine prior to their posting in the outskirts by the Ministry of Health.

The teaching/learning materials for forensic medicine have varied over the ages. At one time the wax models depicting the various ‘Faces of Death’ have now been replaced by instant photos of many cases that are now more readily available. As far as books are concerned the first one written in Nepal was by an American professor who worked for a decade as faculty at the IOM. The second was by an Indian professor who was at one time a faculty at Kathmandu University.

1. Death & Deduction. A. Jay Chapman. Prof. of Forensic Medicine, IOM.
2. Forensic Medicine & Toxicology. BK Sharma, Prof. of Forensic Medicine, Kathmandu University.
3. Chikitsha Bidhishastra (Medical Jurisprudence) in Nepali. Ekraj Acharya

Perhaps new books on Forensic Medicine need to be written as this is an important area where rapid advances are taking place. A lot needs to be done for the development of Forensic Medicine in Nepal especially in view of the fact that there are now new laws being implemented in this federal republic of ours.
